# Type VI collagen promotes lung epithelial cell spreading and wound-closure

**DOI:** 10.1371/journal.pone.0209095

**Published:** 2018-12-14

**Authors:** Jared A. Mereness, Soumyaroop Bhattacharya, Qian Wang, Yue Ren, Gloria S. Pryhuber, Thomas J. Mariani

**Affiliations:** 1 Division of Neonatology and Pediatric Molecular and Personalized Medicine Program, Department of Pediatrics, University of Rochester, Rochester, New York, United States of America; 2 Department of Biomedical Genetics, University of Rochester, Rochester, New York, United States of America; Cedars-Sinai Medical Center, UNITED STATES

## Abstract

Basement membrane (BM) is an essential part of the extracellular matrix (ECM) that plays a crucial role in mechanical support and signaling to epithelial cells during lung development, homeostasis and repair. Abnormal composition and remodeling of the lung ECM have been associated with developmental abnormalities observed in multiple pediatric and adult respiratory diseases. Collagen VI (COL6) is a well-studied muscle BM component, but its role in the lung and its effect on pulmonary epithelium is largely undetermined. We report the presence of COLVI immediately subjacent to human airway and alveolar epithelium in the pediatric lung, in a location where it is likely to interact with epithelial cells. In vitro, both primary human lung epithelial cells and human lung epithelial cell lines displayed an increased rate of “wound healing” in response to a scratch injury when plated on COL6 as compared to other matrices. For the 16HBE cell line, wounds remained >5-fold larger for cells on COL1 (p<0.001) and >6-fold larger on matrigel (p<0.001), a prototypical basement membrane, when compared to COL6 (>96% closure at 10 hr). The effect of COL6 upon lung epithelial cell phenotype was associated with an increase in cell spreading. Three hours after initial plating, 16HBE cells showed >7-fold less spreading on matrigel (p<0.01), and >4-fold less spreading on COL1 (p<0.01) when compared to COL6. Importantly, the addition of COL6 to other matrices also enhanced cell spreading. Similar responses were observed for primary cells. Inhibitor studies indicated both integrin β1 activity and activation of multiple signaling pathways was required for enhanced spreading on all matrices, with the PI3K/AKT pathway (PI3K, CDC42, RAC1) showing both significant and specific effects for spreading on COL6. Genetic gain-of-function experiments demonstrated enhanced PI3K/AKT pathway activity was sufficient to confer equivalent cell spreading on other matrices as compared to COL6. We conclude that COL6 has significant and specific effects upon human lung epithelial cell-autonomous functions.

## Introduction

There are 28 known families of collagens, with subtypes based on function and structure; fibrillar, FACIT (Fibril Associated Collagens with Interrupted Triple helices), beaded filament, anchoring fibril, transmembrane and network forming collagens [1]. Fibrillar collagens I and III are the most abundant collagens in the lung parenchyma and provide most of the structure to the alveolar wall [2]. Several other collagens represent essential components of the lung ECM, including COL6, which can be found in the basement membrane in the lung parenchyma, airways and vasculature [3, 4]. The basement membrane is a specialized ECM structure that separates the epithelium, mesothelium and endothelium from underlying cells and connective tissue. It has been shown that deposition of the basement membrane and other ECM components is a critical event in alveolar septation during lung development [5]. By classical definition, the basement membrane is composed of collagen IV, laminin and entactin, and interacts with other collagens, heparin sulfate proteoglycans (HSPGs) and many other ECM components [6, 7]. Alterations in extracellular matrix composition and the expression of basement membrane components have been shown in many pulmonary disorders, including bronchopulmonary dysplasia (BPD), asthma, chronic obstructive pulmonary disorder (COPD) and idiopathic pulmonary fibrosis (IPF) [8].

COL6 is a hetero-trimer composed of protein products of 6 distinct genes (*COL6A1-6*). COL6 is a beaded filament-forming collagen typically found associated with the basement membrane [9, 10]. It has been observed to form a lattice-like structure that functions to link cells with other basement membrane components [11, 12]. Previous studies have reported the presence of COL6 in the basement membrane of the airway and respiratory epithelium as well as the vasculature in humans and mice [13, 14]. Two mouse models of COL6 deficiency, due to targeting either Col6a1 or Annexin A2 show altered lung physiology, with indiciations of abnormalities in epithelial cell homeostasis [13]. Diseases associated with COL6 include Bethlem Myopathy and Ullrich congenital muscular dystrophy (UCMD) [15]. It has been shown that patients with UCMD display a rapid decline in respiratory function early in disease progression relative to Duchenne muscular dystrophy, which does not directly involve COL6 [16]. Though it has not been thoroughly studied in pulmonary biology, COL6 has also been linked with pathological findings in pulmonary fibrosis and with pulmonary elasticity [2, 15, 17, 18]. Together, these data establish COL6 as a molecule with physiological and pathophysiological relevance to the respiratory system.

The lung epithelium is composed of many distinct cell types including ciliated, goblet, basal, and alveolar epithelial type I and type II cells, rests upon and interacts with the basement membrane [19–22]. Many developmental processes are dependent upon changes in epithelial cell activity to occur properly. For example, the processes of branching morphogenesis and alveogenesis require extensive epithelial proliferation to drive the expansion of the tissue [5, 23]. The composition of the ECM has a strong influence on the phenotype and activity of adjacent epithelial cells. For instance, signaling for proliferation, survival, injury response and migration of lung epithelial cells during and post development can be directed by the ECM [24–34]. Many of these ECM interactions and processes are mediated by integrins, commonly acting through intracellular signaling molecules Rho and Rac[35–39]. The ECM has also been known to selectively sequester, release, and present growth factors and cytokines that can also impact epithelial function [6, 25, 28, 40–42]. Models of alveolar and bronchial epithelium are well suited for studying environmental exposures and aspects of signaling, proliferation, and differentiation[43, 44]. Non-transformed human bronchial epithelial cells (NHBE), and the transformed 16HBE bronchial epithelial cell line, differentiate and express proteins associated with multiple epithelial cell types when cultured at air-liquid interface (ALI) [45, 46]. These cell types represent physiologically relevant tissue models that can be used to assay various components of lung growth and homeostasis that may be affected in disease. Due to the intimate connection between epithelial cells and the development and maintenance of the lung, the epithelium is often critically involved in respiratory disorders, including COPD, BPD and interstitial lung diseases [47, 48]. Therefore, reduced proliferation or ability to maintain barrier function of lung epithelium may lead to aberrant development or injury repair [49, 50]. This study provides novel data on the function of a poorly understood component of the lung basement membrane and its potential role in supporting lung epithelial cell phenotype and function.

## Methods

### Immunofluorescent staining

Formalin fixed, paraffin embedded lung tissue sections were obtained from the human tissue core of the lung development molecular atlas program (LungMAP) at the University of Rochester. Slides were rehydrated in histology xylene (2x) (, 1:1 xylene:100% ethanol (Koptec, King of Prussia, PA, 89125–188), 100% ethanol (2x), 95% ethanol (Koptec, 89125–180), 70% ethanol, 50% ethanol, tap water for 3 minutes each. Antigen retrieval was performed for 20min in Sodium citrate buffer made with 10nM sodium citrate (EMD Millipore, Darmstadt, Germany, SX0445-1), 0.05% Tween 20 (Thermo Scientific, Waltham, MA, USA, 23336–2500) at pH 6.0 in a vegetable steamer at 95°C, and rested for 10min under cool, running tap water. Slides were then washed in Tris-buffered saline (TBS) made with 6.05g Tris (IBI Scientific, Peosta, IA, IB70144), and 8.76g Sodium Chloride (Acros, 42429–5000) with 0.025% Triton X-100 (Fisher Scientific, Hampton, NH, BP151-100) and blocked in 10% normal Goat Serum (Abcam, Cambridge, MA, ab7481) with 1% bovine serum albumin (BSA) (EMD Millipore, 81-068-5) in TBS for 2hr at room temperature. Primary antibody for Collagen VI (Abcam, ab6588), or normal rabbit IgG (Santa Cruz Biotechnology, Dallas, TX, sc-2027) was diluted 6μg/ml in TBS with 1% BSA and applied to slides, which were kept at 4°C overnight. Slides were rinsed with TBS with 0.025% Triton X-100, and FITC-conjugated fluorescent secondary antibody (Thermo Scientific, a11034) was diluted to 4μg/ml and applied to slides for 1hr at room temperature. Finally, slides were washed with TBS with 0.025% Triton X-100, drained, and coverslips were mounted with Prolong Gold antifade mounting medium with DAPI (Thermo Fisher, P36935). Images were taken at 40x magnification using a Leica DFC365FX Camera mounted to a Leica DM5500B microscope and controlled by Leica Advanced Fluorescence version 3.1.0 imaging software (Leica, Wetzlar, Germany).

### Cell culture

16HBE cells, a large-T antigen transformed human bronchial epithelial cell line, were cultured under standard conditions in 37°C in a humidified incubator containing 5% CO2, using DMEM (Gibco, Gaithersburg, MD, cat. 11965092) medium supplemented with 10% FBS (Gibco, 10082147), 1% penicillin/streptomycin (Gibco, 15140122), 1% nonessential amino acids (Gibco, 11140050), sodium pyruvate (Gibco, 11360070), and HEPES buffer (Gibco, 15630080). Media was replaced every 48 hours.

Primary human bronchial epithelial cells, NHBE, (Lonza, Basel, Switzerland, CC-2540S) were cultured under standard conditions in 37°C in a humidified incubator containing 5% CO2, and were used between passages 2 and 3. Cells were expanded in bronchial epithelial basal medium containing bovine pituitary extract (BPE), hydrocortisone, human recombinant epidermal growth factor, epinephrine, insulin, triiodothyronine, transferrin, gentamicin, amphotericin B, retinoic acid, and BSA (Lonza BEGM Bullet kit, CC-3170) supplemented with additional BPE to a final concentration of 130μg/ml (Lonza, CC-4009), Bovine Serum Albumin to a final concentration of 1.5 μg/ml (Gibco, 15260), retinol-al-trans to a final concentration of 1.2e^-7^M (Sigma Aldrich, St. Louis, MO, R7632), and additional hEGF to a final concentration of 25 ng/ml (Sigma Aldrich, E9644). For growth of cells after the first passage, the basal medium was modified to a 1:1 mixture of BEBM/DMEM with high glucose (Gibco, 11965092) containing the same supplements, without additional hEGF (0.5 ng/ml). Media was replaced every 48 hours.

Primary pediatric human lung epithelial (PHLE) cells were isolated from distal lung tissue digests of donor-quality normal lung tissue from 1-day, 2-month and 9-month old donors which were obtained by the human tissue core of the lung development molecular atlas program (LungMAP; lungmap.net) at the University of Rochester. Lung tissue was digested with a protease cocktail containing collagenase (Sigma-Aldrich, St. Louis, MO), dispase (Corning, Corning, NY), elastase (Worthington-Biochem, Lakewood, NJ) and DNAase (Sigma-Aldrich). Dissociated cells were washed twice in DPBS containing 1% Penicillin-Streptomycin (Gibco), 50 μg/ml Gentamicin and 0.25 μg/ml amphotericin B and centrifuged with 800 xg for 10 minutes [51]. These cells were then expanded in small airway basal medium containing bovine pituitary extract, hydrocortisone, human recombinant epidermal growth factor (0.5 ng/ml), epinephrine, insulin, triiodothyronine, transferrin, gentamicin, amphotericin B, retinoic acid, and BSA (Lonza SAGM Bullet kit, CC-3118) and supplemented with 1% FBS. Only 5% of cells in initial cultures are positive for pan-cytokeratin. When 60% confluent, the cultures were gently trypsinized at room temperature with 2ml of 0.0125% trypsin EDTA (Gibco, 25200056) to remove fibroblasts. After depletion of fibroblasts, 95% of cells in these cultures stain for pan-cytokeratin and nearly 70% are positive for both EpCAM and SCGB1A1, indicating that the cultures are highly enriched in epithelial-like cells. By qRT-PCR, PHLE cells express markers of airway epithelium. These expression data suggest that PHLE cells most closely resemble distal airway epithelium [51]. All experiments were conducted using PHLE cells between passages 1 and 3.

### Extracellular matrix coatings

Tissue culture plates were coated with either COL6 from human placenta (Rockland, Limerick, PA, 009-001-108), rat tail collagen I (BD Biosciences, San Jose, CA, 354236), growth-factor depleted Matrigel (Corning, Corning, NY, 356231), or combinations of Matrigel and type I or COL6. Matrices were diluted in phenol red-free DMEM (Gibco, 31053028) to a final concentration of 0.44 nM. For conditions in which Collagen I or VI were mixed with Matrigel, the final mixture was comprised of 0.22nM (1:1 ratio) of each component, for a final concentration of 0.44nM. *In vitro* spreading assays were performed in 48-well plates (containing 50μl of diluted matrix per well), while wound-healing assays were performed in 12-well plates (containing 150 μl of diluted matrix per well). After application of matrix solutions, plates were covered and kept at 37°C overnight, and uncovered in the hood to dry prior to use.

### Wound-healing experiments

Cells were plated on coated 12-well plates at 300,000 per well and grown to confluence (72-96hr). The monolayer was scratched vertically in each well with a 10μl pipette tip. Images of the scratch were taken at consistent locations using 10x magnification immediately after the scratch (0hr), and 6hr, 10hr and 24hr after the scratch. Wound-width was measured in 3 locations and averaged for each image at each time point. Images were taken using an Olympus IX71 inverted microscope connected to an Olympus DP70 Camera operated with DP Olympus Manager software (Olympus, Center Valley, PA, USA)

### Cell spreading experiments

Cells were plated at 50,000 cells per well in matrix-coated 48-well plates. Three images of different locations in each well were taken at 10x power immediately after plating (0hr), 1hr, 2hr, 3hr and 5hr post-plating. All cells in each acquired field (minimum of 200 total spread and unspread cells) were counted using the ImageJ Cell Counter plugin (National Institutes of Health, Bethesda, MD, USA) software. Classification of cells as spread or unspread was performed as described previously [52–54]. Unspread cells retain a uniformly rounded shape with clearly defined cellular borders that are highly refractory under light microscopy. There is also no visible nucleus or obvious organization of the cytoplasm. Spread cells appear much larger and more irregular in shape than unspread cells due to the presence of extended filopodia and lamellipodia. Because of the flattening and extension of the cytoplasm, the cell borders are less refractory and less visible, while the nuclei are more easily identified. The reported percentage of spread cells is calculated by dividing the number of spread cell by the total number of cells counted.

### Integrin and NG2 blocking experiments

For spreading assays, cells were treated with β1-integrin blocking antibody (Abcam, Cambridge, UK, ab24693) or an anti-NG2 antibody that targets the functional domain of NG2 (Abcam, ab78284) in serum-free medium for 5 minutes prior to plating. Antibodies remained present in culture media for the duration of the assays. Both antibodies were diluted 1:500 to a concentration of 2 μg/ml.

### Inhibitor experiments

Inhibitor experiments were performed in serum-containing medium, and treatments began 5 minutes prior to plating (spreading) or 5 minutes prior to scratch-wounding. Small-molecule inhibitors against signaling proteins, CDC42 (ZCL 278, 55 μM in DMSO), ERK (FR 180204, 3 μM in DMSO), FAK (PF 573228, 40 nM in DMSO), PI3K (LY294002, 5 μM in DMSO), RAC (EHT 1864, 600 nM in DMSO), and RHOA (CCG 1423, 3 μM in DMSO) were obtained from Tocris Bioscience, Bristol, UK. In order to ensure specificity of the inhibitors, dose-range studies were conducted with 3 doses for each inhibitor. Concentrations were chosen based on the lowest concentration for significant effect on spreading on any matrix. All inhibitors were used at a concentration less than 5x of the reported In vitro ID50 for each molecule.

### Gain-of-function experiments

Plasmid constructs containing constitutively active mutants of signaling molecules were transfected into 16HBE in reduced-serum Opti-MEM medium (31985070,Gibco). Cells were seeded at 5.0e^5^ cells per well of a 6-well plate. At 18 hours after seeding, when cells are around 50% confluent, medium was removed, wells were washed with PBS, and 700 μl Opti-MEM was added to each well. For each well, 9 μl Lipofectamine 2000 transfection reagent (Invitrogen, Carlsbad, CA, 11668027) was added to 150 μl Opti-MEM. A total of 3.5 μg pDNA was added to a separate tube containing 150 μl Opti-MEM. The diluted transfection medium was added to the diluted pDNA and mixed by vortexing. After 5min incubation at room temperature, 300 μl of this mixture was added to 1 well of the plate. Transfections were performed in duplicate wells. Plasmids containing GFPmPA-GFP-N1 (a gift from Michael Davidson, Addgene plasmid # 54712), and constitutively active FAK (pGFP FAK Y397F, a gift from Kenneth Yamada, Addgene plasmid # 50516), CDC42 (a gift from Joan Brugge, Addgene plasmid #14568), and RAC1 (pcDNA3-EGFP-RAC1-Q61L, a gift from Gary Bokoch, Addgene plasmid #12981) all contain GFP and were transfected individually [55, 56]. Plasmids containing constitutively active PI3K (pBabe puro Myr HA PIK3CA, a gift from Jean Zhao, Addgene plasmid #12523), ERK (pCMV-myc-ERK2-L4A-MEK1_fusion, a gift from Melanie Cobb, Addgene plasmid #39197) and RHOA (pCDNAIIIB RHOA QL, a gift from JS Gutkind, Addgene plasmid #61632) do not express fluorescent markers and were transfected at a ratio of 10:1 signaling construct:GFP construct in order to visualize transfected cells.[57–59]. Each construct, aside from GFP expresses a signaling molecule that has been modified by mutation to a phosphomimetic residue or introduction of a myristoylation site to be constitutively active. All plasmids were purchased from Addgene, Cambridge, MA, USA. Transfections were performed for 36 hours, after which transfection medium was removed, wells were washed with PBS, and 2 ml fresh growth medium was added. After 12 hours of recovery, spreading assays were performed with transfected cells as described previously, counting only transfected, GFP-positive cells in order to explicitly quantify the spreading response in cells expressing the constitutively active signaling molecules. As controls, cells were transfected with the mPA-GFP-N1 construct expressing GFP, in order to test spreading in only cells that have undergone transfection. Both FITC fluorescence and brightfield images were taken at 10x magnification in 3 separate fields per well. Using ImageJ software, green fluorescence was merged with the brightfield images, and spreading status of only fluorescent cells was determined.

### Statistical analysis

All tests of significance were performed using a 2-tailed, unpaired t-test implemented in Microsoft Excel 2010 (Microsoft, Redmond, WA).

## Results

### COL6 expression in human lung tissue

**Fig 1:**
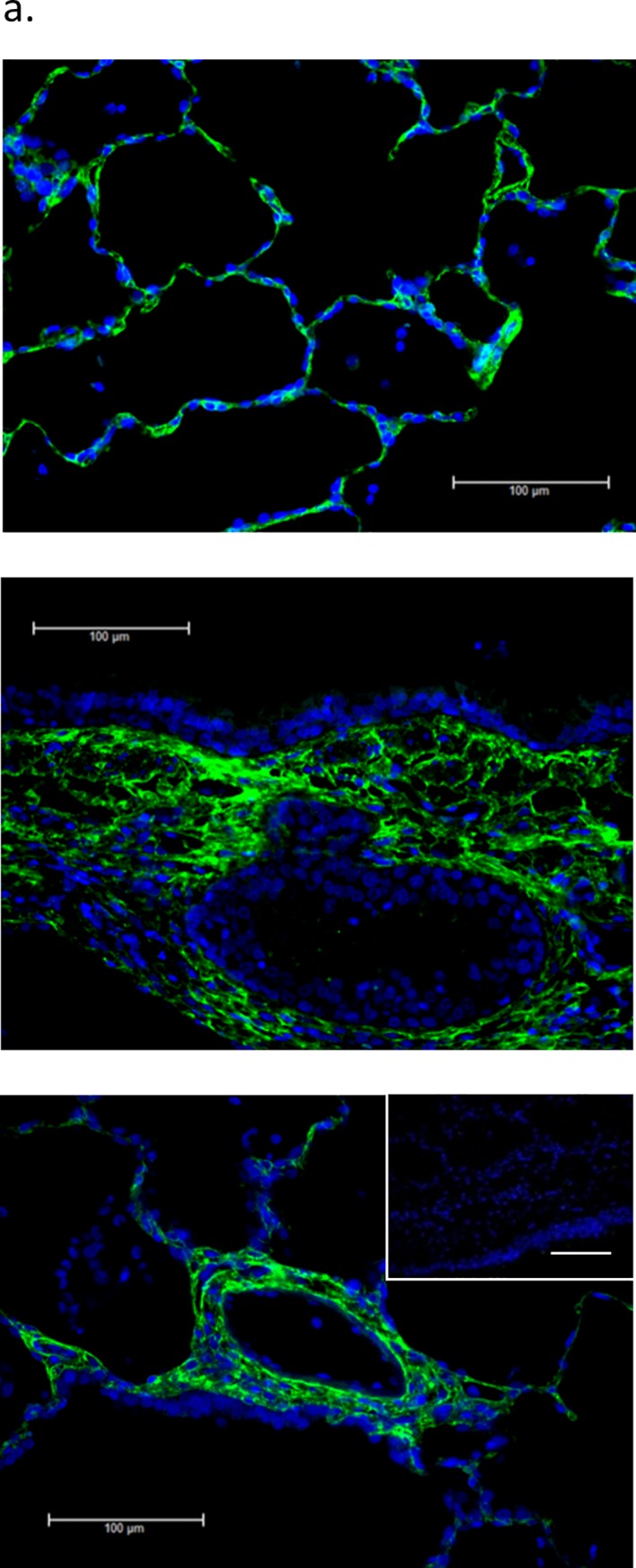
COL6 is present in the basement membrane region throughout the lung. (a) Immunohistochemical staining of COL6 (green) in normal human lung tissue shows deposition of this extracellular matrix molecule in the basement membrane of large and small airways, the alveolar parenchyma and the vascular wall. Nuclei are counter-stained blue. All scale bars are 100μm. Inset represents IgG control.

Prior publications have reported COL6 expression in the lung of the mouse and pig [14, 60]. We sought to confirm similar localization in the human lung, particularly prior to maturity. We performed immunohistochemistry on fixed, human pediatric lung tissue sections obtained from the developing lung molecular atlas program (LungMAP). As seen is Fig 1, Collagen VI is broadly detectable in the basement membrane region, beneath the airway epithelium and vascular endothelium, as well as throughout the lung parenchyma.

### COL6 promotes epithelial cell wound healing

**Fig 2:**
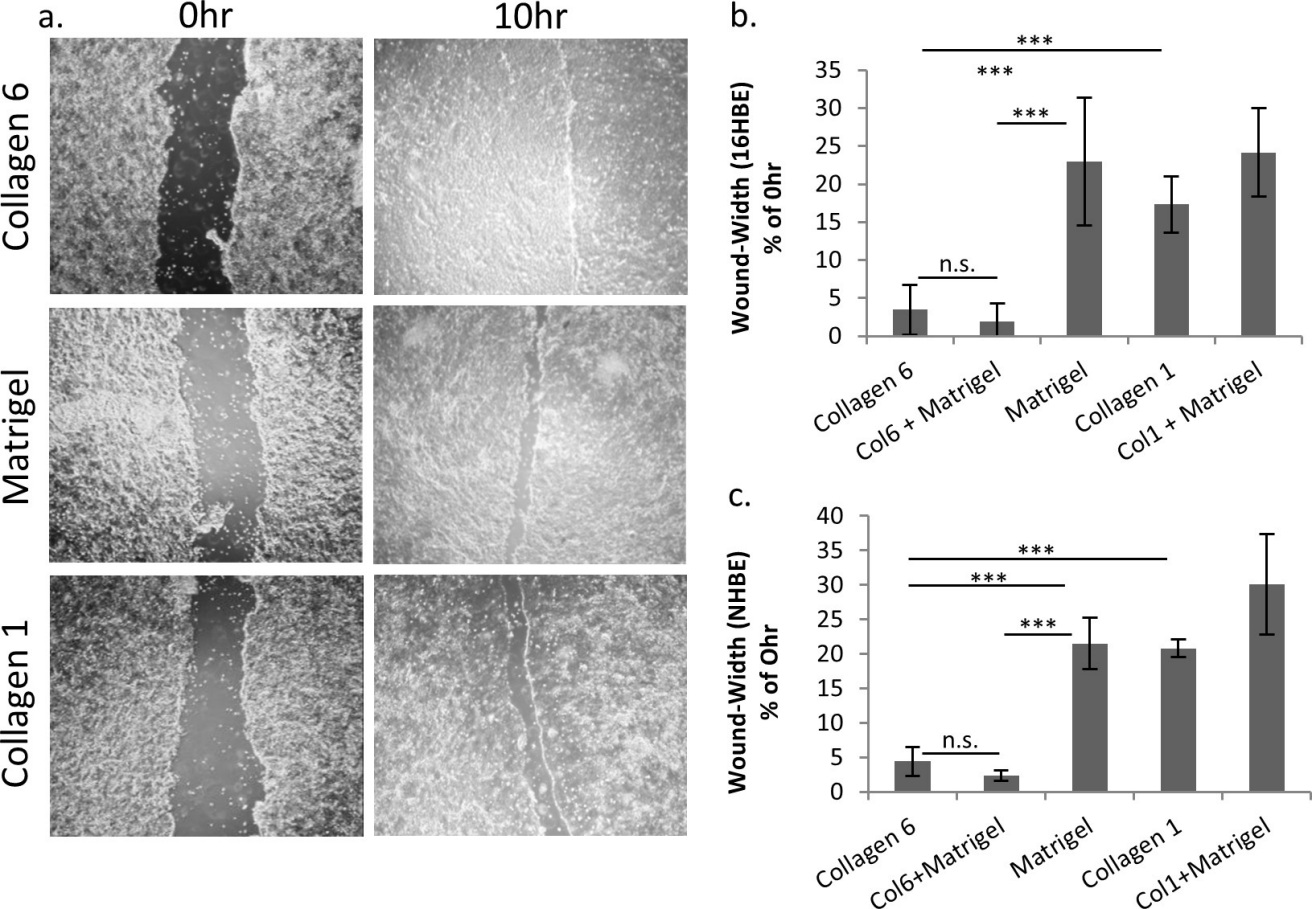
COL6 enhances lung epithelial cell wound repair. (a) Representative images of wound-healing response for 16HBE cells plated on COL6, Matrigel, or COL1. (b-c) Quantitation of wound width at 10 hr post-injury (relative to 0 hr) for 16HBE(b) and NHBE(c) plated on individual matrices, or combinations of matrices. N = 9, ** p<0.01, *** p<0.001.

The aberrant injury response in many developmental and chronic lung diseases suggests an impairment of the injury repair process. Therefore, the effect of COL6 and other matrices on wound-healing response of the epithelium was tested using a standard *in vitro* monolayer scratch assay, as a method for measuring monolayer migration. Previous experiments showed no difference in cell proliferation on the matrix molecules studied (S1 Fig, S1 Text). As shown in Fig 2A and 2B, bronchial epithelial cells (16HBE) show a significant reduction in remaining wound width on COL6 (3.4% of 0 hr control) relative to Matrigel (23.0%, p<0.001) or COL1 (17.3%, p<0.001) 10 hours after scratch. This was consistent with observations using primary bronchial epithelial cells (NHBE), which showed a significantly smaller wound width on COL6 (4.4%) compared to Matrigel (21.5%, p<0.001) and COL1(20.8%, p<0.001) (Fig 2C). Wound healing and spreading responses were next characterized on matrices containing combinations of COL6 or COL1 with Matrigel, which contains many of the common basement membrane constituents. Matrices combining COL6 with Matrigel promoted wound healing to a level that is not significantly different from wound healing on COL6 alone. At 10hr post-scratch, the wound width of 16HBE cells on COL6 and COL6 mixed with Matrigel was not significantly different. The wound width on both COL6 and Matrigel with COL6 (2.0%) was significantly less than on Matrigel alone (p<0.001) and COL1 alone (p<0.001). Conversely, the wound width of cells on COL1 with Matrigel (24.2%) was not significantly different than that on COL1 or Matrigel alone (Fig 2B).

Normal human bronchial epithelial cells (NHBE) showed similar wound healing responses. The wound width on both COL6 and Matrigel with COL6 (2.3%) was significantly less than on Matrigel alone (p<0.001) and COL1 alone (p<0.001). The wound width of NHBE cells on COL6 alone, or COL6 mixed with Matrigel was not significantly different. The wound width of cells on COL1 with Matrigel (30.1% vs. 0hr wound-width) was not significantly different than that on COL1 or Matrigel alone (Fig 2C).

In addition, wound healing assays were carried out with primary human lung fibroblasts. The wound width on matrigel (2.7%) was significantly less than the wound with on COL6 (36.7%, p<0.05) and COL1 (45.6%, p<0.05) (S3 Fig, S2 Text).

### COL6 promotes epithelial spreading

**Fig 3:**
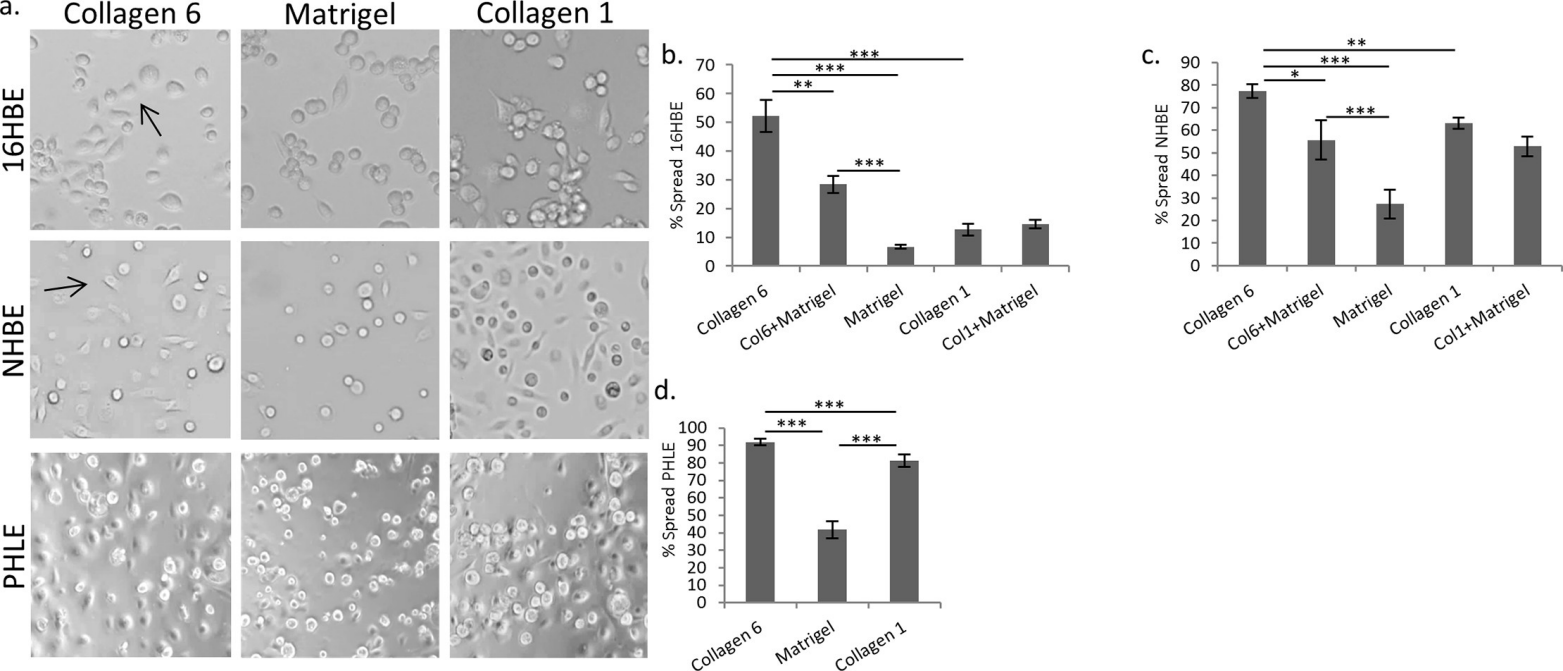
COL6 promotes lung epithelial cell spreading. (a) Representative images of initial, post-adhesion spreading for 16HBE human lung epithelial cells 3 hours after plating cells on COL6, COL1 or Matrigel. Arrows show examples of spread cells. (b-d) Quantitation of the percentage of cells spread 3 hours after plating on different matrices for 16HBE (b), NHBE (c), or PHLE (d) cells. N = 6.* p<0.05, ** p<0.01, *** p<0.001.

In order to examine a less complex aspect of the sophisticated wound-healing response, the kinetics of the spreading response were tested. Previous experiments had determined that there was no difference in the ability of cells to adhere to COL6, Matrigel or COL1 (S2 Fig, S1 Text). We tested whether cell spreading may play a role in the observed wound-healing response. Indeed, 16HBE spreading kinetics were significantly impacted by the presence of COL6. The percentage of spread 16HBE cells (52.2%) on COL6 at 3 hours after plating is nearly 8-fold higher than on Matrigel (6.7%, p<0.001) and over 4-fold higher than COL1 (12.6%, p<0.001) (Fig 3A and 3B). Similarly, the percentage of NHBE cells spreading on COL6 (77.3%) was greater than 2-fold higher than on Matrigel (27.3%, p<0.001) and was significantly greater than COL1 (63.0%, p<0.001) (Fig 3C).

We next tested primary pediatric human lung epithelial (PHLE) cells for their response to COL6, since they represent an age that is developmentally and physiologically distinct from the adult-derived NHBE cells. PHLE cells showed a spreading response similar to other pulmonary epithelial models on the tested matrices. There were greater than 2-fold more spread cells on COL6 (91.9%) at 3 hours than Matrigel (41.8%, p<0.001) and significantly more than on COL1 (81.4%, p<0.001) (Fig 3D).

We again tested combinations of matrix substrates for their ability to affect epithelial cell spreading rates. Addition of COL6 to Matrigel resulted in an increased percentage of spread cells as compared to Matrigel alone in 16HBE (28.4% vs 6.7%, respectively, p<0.001). However, 16HBE spreading on combined COL6 and Matrigel was significantly less than on COL6 alone (52.2%, p<0.01). There was no significant difference in spreading between cells on COL1 (12.6%) or COL1 combined with Matrigel (14.5%). However, both showed an increase in spreading compared Matrigel alone (p<0.01, Fig 3B). Combining COL6 with Matrigel showed increased percentage of spread NHBE cells over Matrigel alone (55.6% vs 27.3%, respectively, p<0.001). However, this was significantly less than cells on COL6 alone (77.3%, p<0.05). There was no significant difference in spreading between cells on COL1 (63.0%) or COL1 combined with Matrigel (52.8%). However, both showed an increase in spreading than Matrigel alone (p<0.001, Fig 3C).

### Integrin β1, but not NG2 mediates spreading on COL6

**Fig 4:**
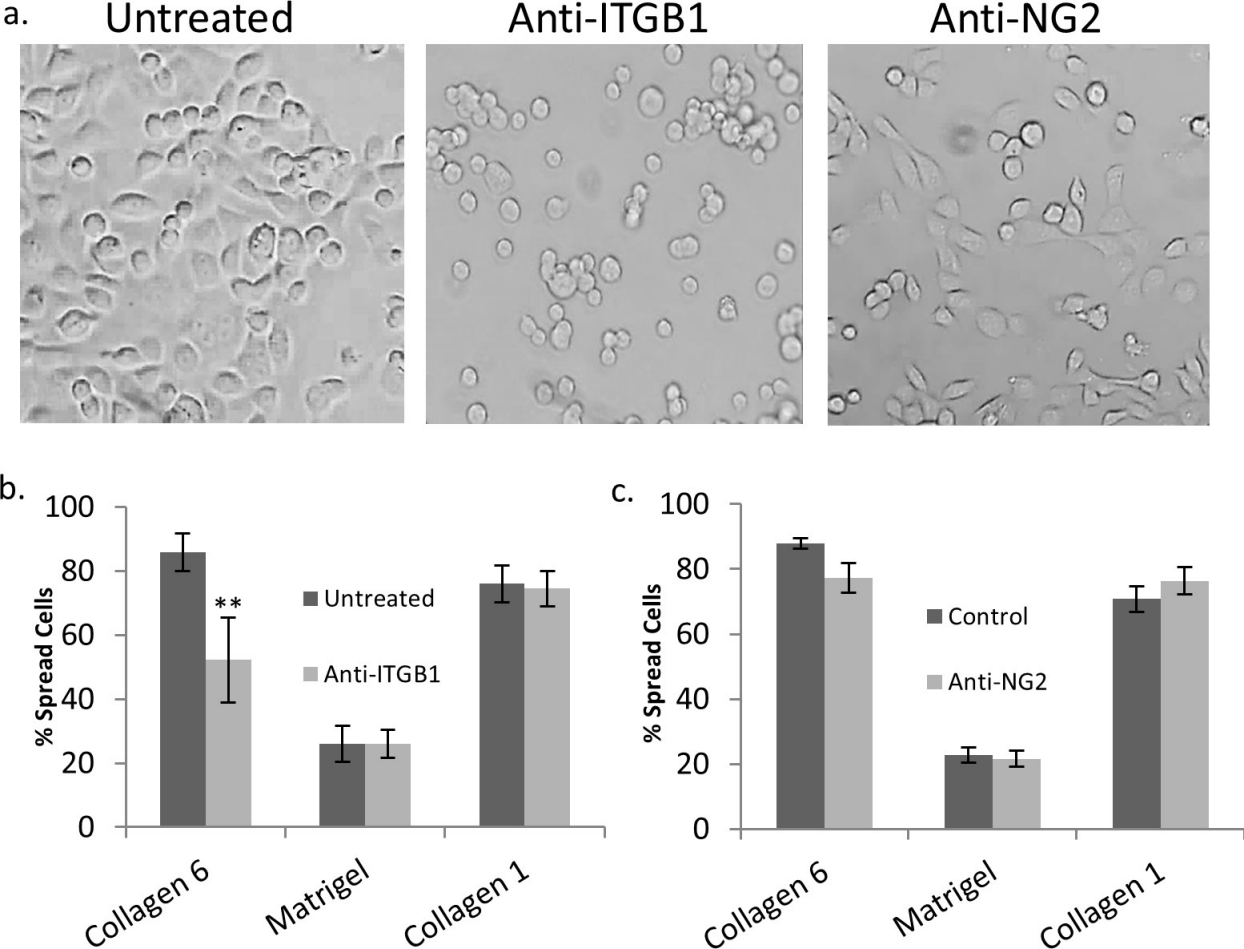
COL6-mediated spreading requires ITGB1, not NG2. (a) Representative images of untreated 16HBE cells, and 16HBE treated with anti-Integrin β1 or anti-NG2 antibodies 3 hours after plating on COL6. (b) Quantitation of untreated and anti-ITGB1 (b) or anti-NG2 (c) treated 16HBE spreading 3 hours after plating on COL6, COL1, Matrigel coated wells, and uncoated tissue-culture wells (n = 6). * p<0.05, ** p<0.01, *** p<0.001.

COL6 is known to interact with β1 integrins and NG2 (CSPG4). This interaction can be independent, or binding with NG2 can cause cross-activation of integrin β1 (ITGB1)[61–65]. In order to test which cell surface receptor for COL6 was responsible for the observed differences in spreading, blocking antibodies were used to inhibit binding to these molecules. The percent of spread 16HBE cells on COL6 (85.8%) was reduced by 40% in the presence of anti-ITGB1 antibody (52.1%)(p<0.01), but anti-ITGB1 antibody did not significantly inhibit spreading on Matrigel or COL1 (Fig 4A and 4B). Inclusion of an anti-NG2 (CSPG4) antibody targeting the active domain of the proteoglycan had no effect on spreading on COL6, Matrigel, or COL1 (Fig 4C).

### COL6 regulates epithelial cell activity via PI3K/AKT signaling

**Fig 5:**
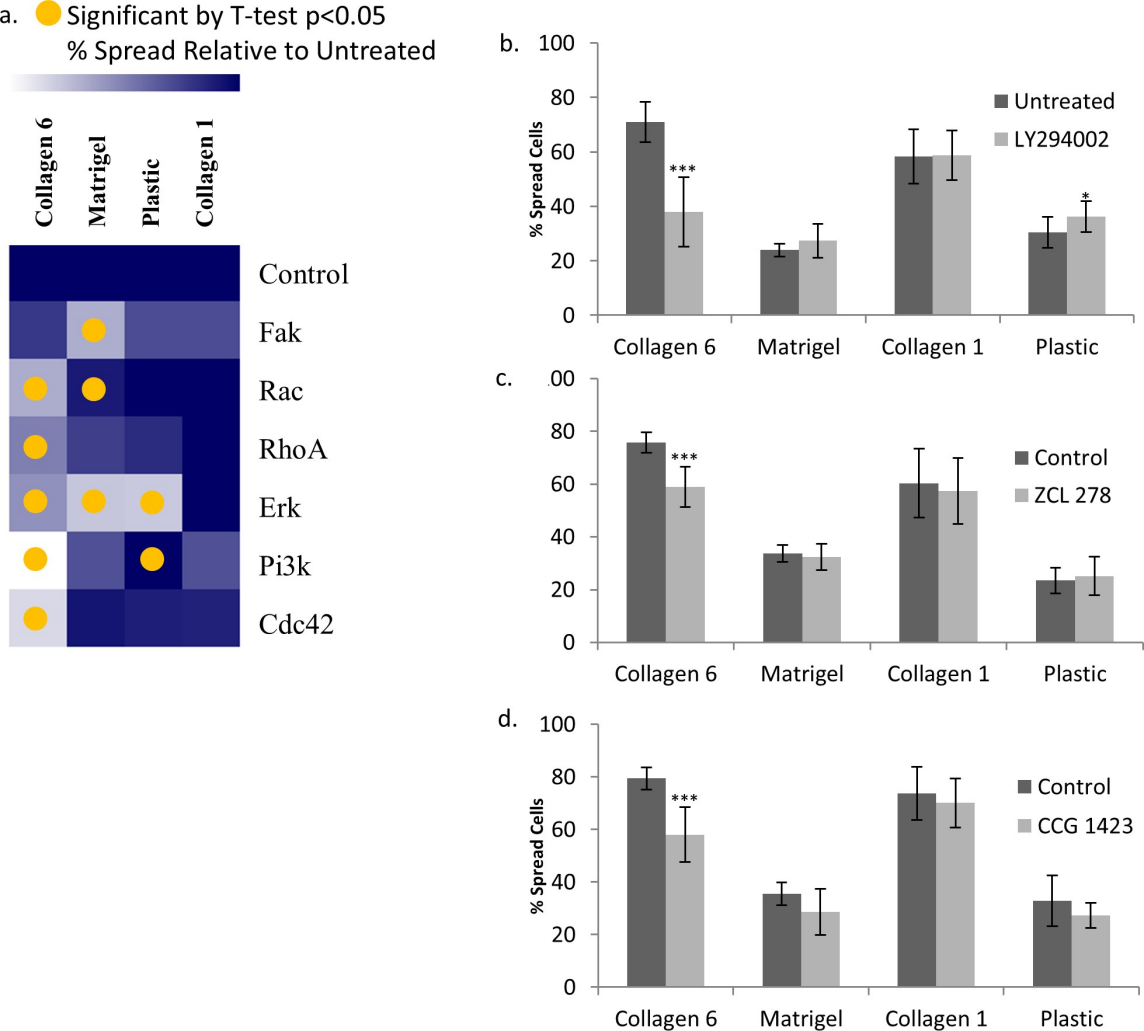
Spreading on COL6 is specifically reduced by inhibiting PI3K, CDC42 and RHOA. Quantification of 16HBE spreading 3 hours after plating on COL6, COL1, Matrigel coated wells, and uncoated tissue-culture wells after treatment with inhibitors ofPI3K (LY294002, 5 μM), CDC42 (ZCL 278, 55 μM), RHOA (CCG 1423, 3 μM), FAK (PF 573228, 40 nM), RAC (EHT 1864, 600 nM), and ERK (FR 180204, 3 μM). (a) Heat map indicating the effect of inhibitor relative to control for cells on each matrix. Lighter color indicates greater effect of the inhibitor on cell spreading on that matrix. A yellow circle represents p<0.05. Quantitation of % spread cells on COL6, Matrigel, COL1, and plastic after treatment with (b) PI3K inhibitor, (c) CDC42 inhibitor, and (d) RhoA inhibitor. * p<0.05, *** p<0.001, N = 6.

We next examined intracellular signaling pathways necessary for enhanced spreading on COL6 by testing the effects of chemical inhibition of key signaling intermediates (Fig 5). Epithelial cell spreading on COL6 was significantly reduced by inhibitors of PI3K (54% of untreated, p<0.001), RAC1 (77%, p<0.001), RHOA (74%, p<0.001), CDC42 (76%, p<0.001) and ERK (76%, p<0.001), but not FAK. Spreading on Matrigel was significantly reduced by inhibitors of ERK (56% of untreated, p<0.001), FAK (64%, p<0.001) and RAC (87%, p<0.05), but not PI3K or CDC42. Spreading on COL1 was not significantly affected by any of the inhibitors at the doses tested. It is important to note that RAC1, CDC42 and RHOA are downstream of PI3K/AKT [66, 67].

**Fig 6:**
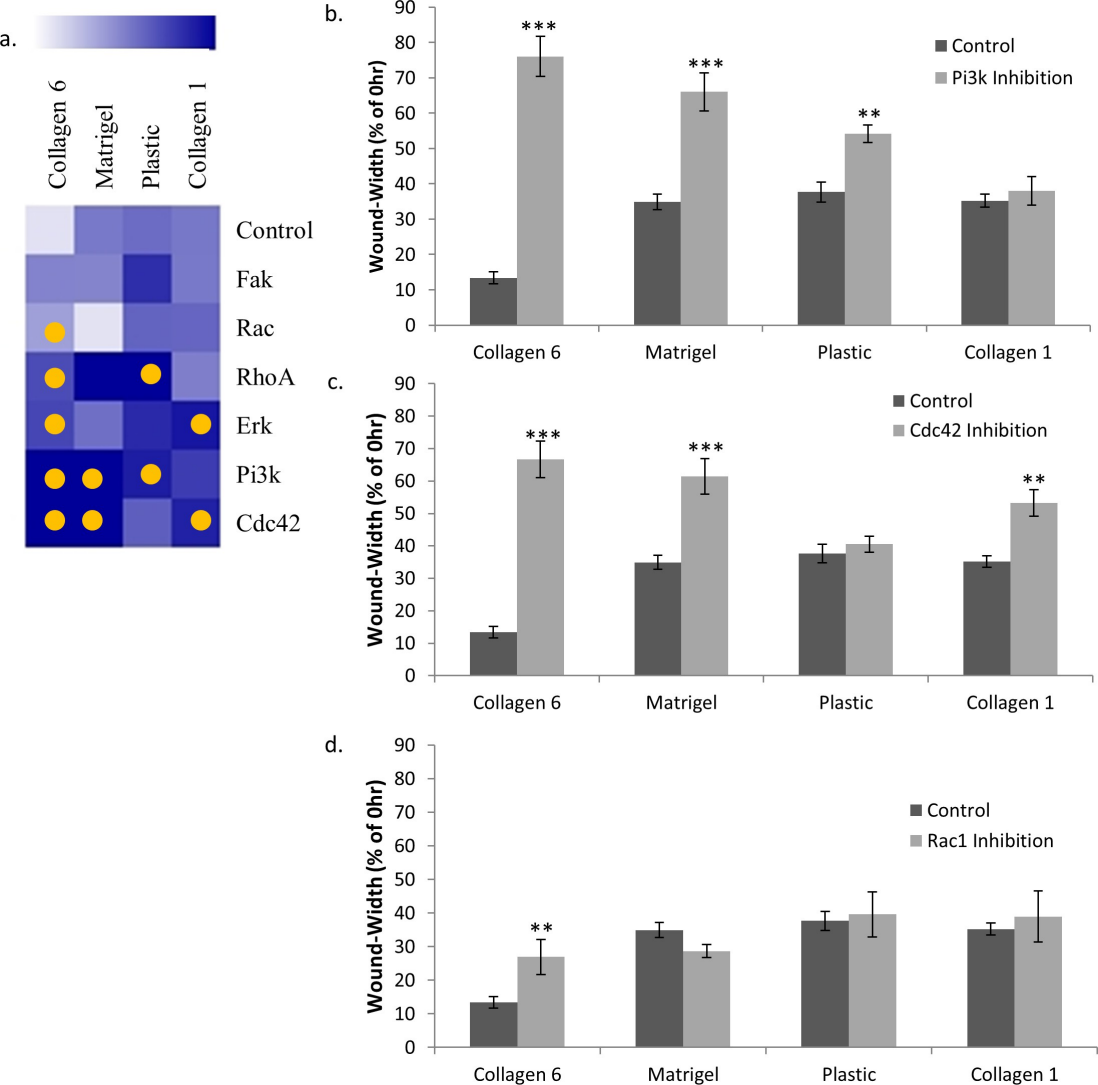
Wound healing on COL6 is specifically slowed by inhibiting RAC1. Quantification of 10hr 16HBE wound-width relative to 0hr controls on COL6, COL1, Matrigel coated wells, and uncoated tissue-culture wells after treatment with inhibitors of PI3K (LY294002, 5 μM), CDC42 (ZCL 278, 55 μM), RHOA (CCG 1423, 3 μM), FAK (PF 573228, 40 nM), RAC (EHT 1864, 600 nM), and ERK (FR 180204, 3 μM). (a) Heat map indicating the effect of inhibitor relative to control for cells on each matrix. A lighter color represents more wound closure relative to 0hr. A yellow circle represents p<0.05. Plots present wound-healing rate of cells on COL6, Matrigel, COL1, and plastic after treatment with (b) PI3K inhibitor, (c) CDC42 inhibitor, and (d) RAC1 inhibitor. ** p<0.01, *** p<0.001. n = 6.

We also investigated the intracellular mechanisms involved in the wound healing response on COL6 (Fig 6). The 10-hour wound-width on COL6 was significantly increased relative to untreated controls (13.4% of 0hr) using inhibitors of PI3K (76.1% of 0hr, p<0.001), CDC42 (66.7%, p<0.001), RHOA (44.3%, p = 0.001), RAC (26.9%, p<0.01), and ERK (45.6%, p<0.001), but not FAK. Wound width on Matrigel was significantly increased by inhibitors of CDC42 (61.4% of 0hr vs 34.9% untreated control, p<0.001) and PI3K (66.0%, p<0.001), but not RAC, RHOA, FAK or ERK. Wound width on COL1 was also significantly increased by inhibition of CDC42 (53.3% of 0hr vs 35.2% untreated control, p<0.01), and ERK (55.5%, p<0.01) but not RACRHOA, CDC42, or FAK.

**Fig 7:**
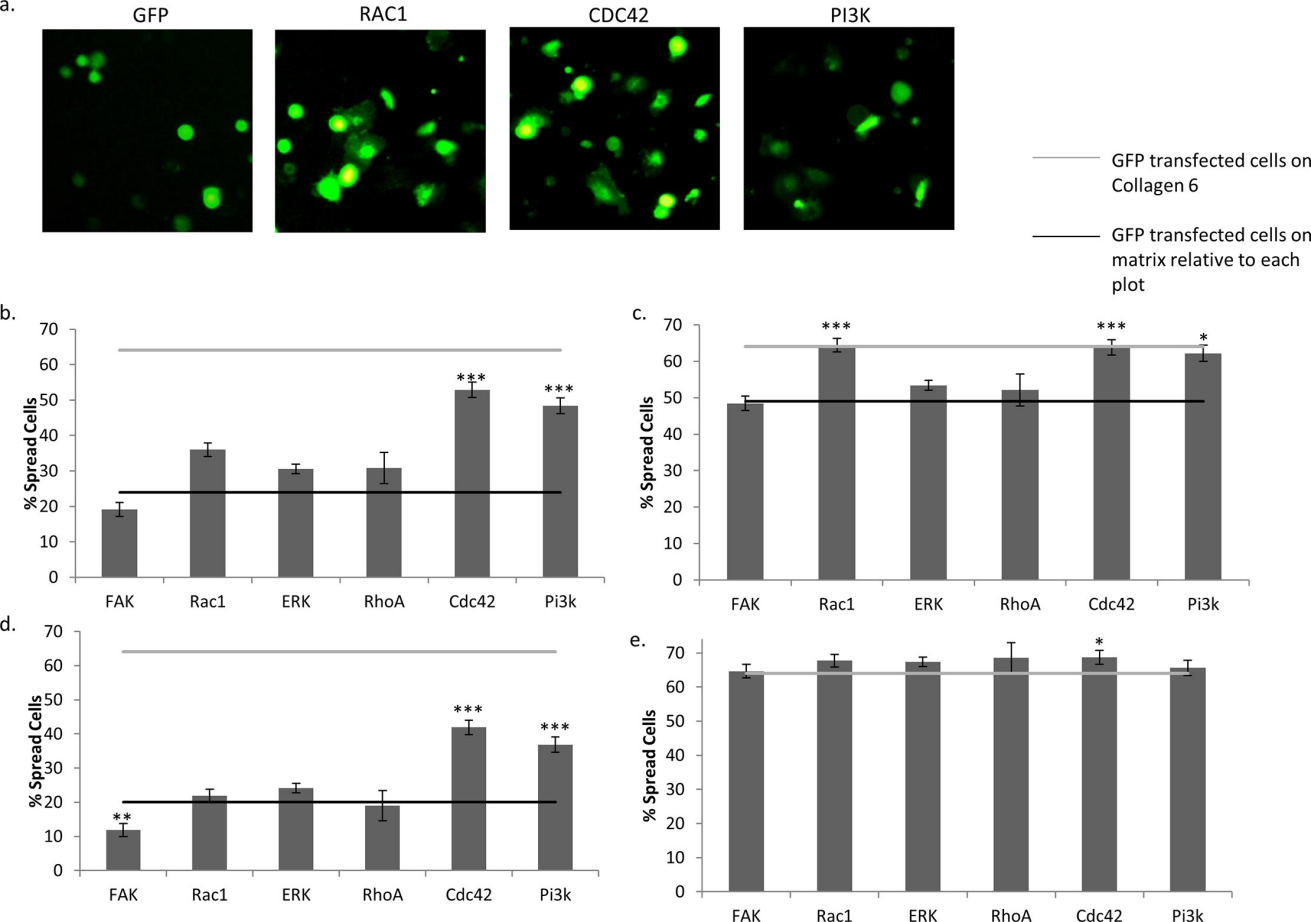
Constitutive activation of PI3K, RAC1 and CDC42 significantly augment spreading in the absence of COL6. (a) Representative images of GFP-only, Rac1, Cdc42 and Pi3k-overexpressing cell spreading on Matrigel. Quantification of GFP-positive 16HBE spreading 3 hours after plating on (b) Matrigel, (c) COL1, (d) uncoated tissue-culture wells, and (e) COL6 after transfection with constitutively active signaling constructs for FAK, RAC1, ERK, RHOA, CDC42, or PI3K. Black lines represent spreading of GFP-transfected cells on each matrix, respectively. Grey lines represent spreading of GFP transfected cells on COL6. N = 6. * p<0.05, ** p<0.01, *** p<0.001.

Finally, as validation of the inhibition results, we attempted to identify signaling intermediates that are sufficient to promote spreading on COL6-deficient basement membrane to the level of spreading on COL6 (Fig 7). This was performed by ectopic expression of constitutively active signaling molecules, followed by assessment of spreading by GFP-positive cells expressing these constructs (see Methods) relative to cells transfected with GFP only. Using this approach, the percentage of cells spread on Matrigel was significantly increased by constitutively active RAC1 (12.0%, p<0.01), CDC42 (28.9%, p<0.001) and PI3K (24.4%, p<0.001) gain-of-function constructs (Fig 7A and 7B). On COL1, cells transfected with RAC1 (15.3%, p<0.001), CDC42 (14.7%, p<0.001) and PI3K (13.1%, p<0.01) constructs also showed a significant increase in spreading (Fig 7C). Cell spreading on COL6 was modestly increased by CDC42 (4.6% increase over control, p<0.05), but not by any other gain-of-function construct (Fig 7D). Interestingly, cells on uncoated tissue culture plastic displayed more rapid spreading when transfected with constitutively active CDC42 (21.9%, p<0.001) and PI3K (16.8%, p<0.001) (Fig 7E).

## Discussion

COL6 appears to be an essential component of many extracellular matrices, primarily localizing within or adjacent to basement membranes, where it appears to function to enhance cell-matrix interactions [29,33,39]. While abnormalities in extracellular matrix (ECM) expression, structure and function have been repeatedly implicated in chronic lung disease (COPD, BPD, IPF), how these ECM changes contribute to disease pathophysiology is poorly understood. Although COL6 is known to have cell-specific pathophysiological effects in other tissues, there is a very limited understanding of how it impacts lung physiology and lung cell biology. It is known that lung tissue is one of the highest expressors of COL6, and limited studies indicate that COL6 deficiency may result in abnormal elastance/compliance and airway epithelial cell dysmorphology [13]. However, the significance and mechanisms of these associations is unexplored. Therefore, we set out to test the cell-autonomous effects of COL6 on lung epithelial cells. Although we were unable to find any effect of COL6 upon cell proliferation or adhesion (S1 and S2 Figs, S1 Text), we found that this ECM molecule substantially enhanced lung epithelial cell spreading and wound closure (Figs 2–7). This is the first report of the specific effects of COL6 on cell autonomous functions of pulmonary epithelial cells and the mechanisms driving these functions. Combined, these observations establish COL6 as a novel component of the lung ECM with potential for involvement in developmental, homeostatic, injury-repair and disease mechanisms.

The airway epithelial cells in mice in a model that is unable to deposit COL6 are reported to be not uniform and columnar in appearance, showing increased evidence for apoptosis, possibly due to reduced adhesion to the basement membranes lacking COL6 [13]. Few studies have been performed to directly examine the effects of COL6 on epithelial cells in any organ. Studies of intestinal epithelium have shown that COL6 is involved in the regulation of cell spreading, RHO signaling, and may also affect the expression of other ECM components [9]. Therefore, we sought to test whether the abnormalities in the lungs of mice deficient in COL6 could be partially explained by similar mechanisms. As we are interested in neonatal lung disease that results, at least in part, from injury responses within the context of organ development, we first tested COL6 for the ability to modify epithelial wound healing responses in a standard *in vitro* assay. The *in vitro* wound-healing assay we used is a validated test of the migratory and wound-healing responses of cells in a monolayer, which is a good approximation of *in vivo* migration of many cell types, including epithelial cells [68]. Further, the wound-healing assay has been used extensively to test the effects of cellular interactions with ECM, and its effects on migration. Our results show that COL6 promotes rapid wound healing relative to Matrigel, COL1 or tissue culture plastic. These effects were similar in primary cultures of human lung/airway epithelial cells, as well as in the 16HBE immortalized human lung epithelial cell line. Wound healing assays were attempted with a model of primary human pediatric lung epithelium (PHLE) cells. However, PHLE cells did not respond to close the “wound” in an epithelial, sheet-like fashion in these assays, precluding quantification of closure. Interestingly, we observed no differences in epithelial cell proliferation on these matrices (S1 Fig, S1 Text). Importantly, the addition of COL6 to Matrigel resulted in the maximal rate of response, supporting the conclusion that COL6 supports optimal wound closure by epithelial cells upon a basement membrane-type ECM. We speculate that insufficiency in COL6 may prevent epithelial cells from forming a proper barrier during development, or from regaining that function after injury.

The wound-healing response is highly complex, involving several cell-autonomous functions, including migration, proliferation and cell spreading. In order to better understand the dramatic effects of COL6 upon lung epithelial cell responses in this complex system, we examined its role in less complex cell-autonomous responses. No differences were observed in cell adhesion or proliferation on these matrices. However, cells on matrices containing COL6 displayed initial post-adhesion spreading at a much higher rate than cells on Matrigel, or COL1. The 16HBE cell line, primary cultures of adult human airway epithelial cells and primary cultures of infant/pediatric human airway epithelial cells all showed enhanced spreading on COL6 matrices. Similar to results in the wound healing assay, combining COL6 with Matrigel is also able to significantly promote cell spreading. The observed effect sizes of 10–20% difference in the % of wound closed are consistent with previously reported wound healing assay results using similar methods and cell types [69, 70]. These data suggest that the presence of COL6 in the matrix may promote wound healing by amplifying the spreading of pulmonary epithelial cells.

Previous work has identified multiple potential mechanisms of COL6 binding and signaling [13, 61, 64, 65]. Both Integrin β1 and the membrane-spanning proteoglycan NG2 (CSPG4) are receptors for COL6, and can act independently to facilitate intracellular signaling following ligation. It has also been shown that NG2 can activate and elicit a response through Integrin β1. While we failed to demonstrate an effect of NG2, our studies indicate that ITGB1 is required to promote spreading on COL6. This likely excludes NG2 signaling independent if ITGB1 as a driver of spreading on COL6, but may not rule out cooperation between NG2 and ITGB1 in response to COL6 binding. The possibility of NG2 or other cell surface proteins acting through ITGB1 could explain the differential activity of signaling molecules and cell autonomous function between cells on COL6 relative to Matrigel or COL1, as both are also known to bind to and activate integrins. We were unable to demonstrate the impact of β1 integrin or NG2 antibody treatment in wound healing assays. This may be due to the fact that under steady-state conditions prior to scratch-wound formation, these cells have already formed focal adhesions and mature interactions with the matrix on the basal surface of the cells. These factors may affect the ability of these antibodies to bind and block the function of their targets.

Multiple integrin pairs have been identified as potential binding partners for COL6, including α1β1, α2β1, α3β1, α10β1 and αvβ3 integrins, though specific interactions between COL6 and these potential binding partners are not well defined [71–73]. In addition, while our data support an “outside-in” integrin signaling mechanism based on the GTPases that appear to be involved, the role of “inside-out” signaling, or a potential interplay between both mechanisms cannot be ruled out [74]. “Inside-out” signaling is often associated with the process of cell adhesion, while it has been shown that both mechanisms have the potential to be involved in cytoskeletal reorganization, which is a key component of cell spreading and migration [75, 76]. Further study is necessary to completely understand the potential role of NG2 in these processes, and provide more a more detail on the specific integrin pairs and integrin signaling mechanism involved in these processes.

We explored the function of numerous signaling pathways and effector molecules downstream of integrin signaling in COL6-mediated spreading and wound healing. Signaling molecules were chosen for study based on canonical association with integrin signaling and known involvement in spreading and migration responses on multiple matrices. The most upstream molecules chosen were FAK and PI3K, which are both potential upstream regulators of ERK, RAC1, CDC42, and RHOA via various signaling pathways [77, 78]. However, FAK does not seem to be a key regulator of the spreading or wound healing response in pulmonary epithelial cells, as neither inhibition nor constitutive activation of FAK affected the spreading response on COL6. Inhibition of PI3K does, however specifically reduce spreading on COL6. In addition, constitutive activation of PI3K and CDC42, are able to increase the rate of spreading on Matrigel, COL1 and plastic to nearly what is observed on COL6, a response also observed with constitutive activation of RAC on COL1. PI3K and its downstream effectors, particularly RAC and CDC42, appear to have a distinct role in the augmented spreading and wound healing response observed on COL6. While additional signaling molecules may be involved in the spreading and migration responses, these experiments focused on the enhanced function observed when these cells interact with COL6. It is possible that the involvement of additional pathways and mechanisms in the wound-healing response are the reason for the apparently broader effects of the inhibitors across matrices in the wound-healing assay.

Extensive studies have been performed to dissect the mechanisms controlling spreading and migration in epithelial cells. Regulation of migration is generally attributed to RHO activity, while spreading is generally regulated by RAC and CDC42, though there is extensive interplay between these molecules. During the process of spreading, RAC is known to control lamellipodia formation, while CDC42 regulates filopodia formation. It has also been shown that the protrusion of filopodia may precede lamellipodia formation in the process of spreading [79]. Additionally, CDC42 has been identified as a possible upstream activator of RAC and RHO, indicating that CDC42 activation and filopodia formation may be key events in the propagation of spreading and migratory responses. Taken together, our data indicate that COL6 may promote spreading via CDC42 and RAC downstream of PI3K activation. It is possible that enhanced CDC42 activation leading to filopodia formation is driving increased activation of RAC and lamellipodia formation when cells bind to COL6. Previous studies have shown that CDC42 is responsible for determining cell polarity during migration in wound healing [79, 80]. Our results indicate that CDC42 may also be a key signaling molecule in the augmented wound healing response observed on matrices containing COL6. Previous work has shown the involvement of p38, JNK and ERK among other signaling molecules involved in epithelial spreading and migration responses, and may work concurrently, or synergistically with PI3K [81–84]. While the results reported here indicate that PI3K and CDC42 are responsible for the enhanced response to COL6, they do not rule out the potential contributions of other signaling pathways, and additional studies of known signaling pathways would be informative.

We acknowledge some limitations of our experiments and interpretations. First, all our experiments were performed using conducting airway epithelial cells of the human lung. Both 16HBE and NHBE resemble bronchial epithelium, while PHLE cells most closely resemble the more distal bronchiolar airway (Q. Wang et. al., manuscript submitted). The basement membrane of both the bronchial and bronchiolar airway does appear to contain COL6, providing justification for the study of cellular interactions with COL6 [4, 13, 14]. However, our results may not be representative of cells in the functional respiratory region. Second, our experiments were not performed at air-liquid interface (ALI) culture, which would represent the most physiological setting for these cells. Using ALI would have been complicated, as many of our assays involved sub-confluent cell cultures. Third, we have not specifically addressed the effect of matrix stiffness on COL6-mediated lung epithelial cell spreading and wound healing. Previous studies have shown that stiffer matrices increase wound healing and spreading rate of both epithelial cells and fibroblasts [85–87]. However, the observation that fibroblasts and epithelial cells differ in wound healing response to the tested matrices indicates that our observations are not solely due to differences in matrix stiffness, and that the response to COL6 is not ubiquitous, but somewhat specific to epithelial cells. It should be noted that we used thin coatings of matrix on plastic for these studies, thereby minimizing potential differences in ECM stiffness. However, additional experiments on the stiffness of COL6-containing matrices, and the subsequent effects of these matrices are warranted, and may provide additional insight into COL6 effects on cell autonomous function. Fourth, we acknowledge the potential for subjectivity in the definition of spreading used in the current studies, which is similar to those previously used by others. Additional measures, such as quantitative assessment of cell area, could complement the data presented here and would provide additional information on the impact of matrix on cell size. Finally, though the majority of COL6 mRNA production is attributed to the mesenchyme, it is possible that the epithelial cells used could produce additional COL6 and may modify the matrix coatings over time [13, 88, 89]. The shortened timeline of the spreading assay was used in an attempt to eliminate the effects of matrix modifications by the epithelial cells. Additional characterization of epithelial cell production of COL6 during the processes of adhesion, spreading and migration are warranted.

In summary, we report novel experimental data indicating that COL6 potentiates optimal lung epithelial spreading and wound injury responses. Decreases in COL6 expression in the lung may precipitate epithelial dysfunction leading to abnormal development, aberrant injury repair or decreased barrier function that can contribute to chronic lung disease. Further studies of the roles for COL6 in lung development, homeostasis and disease are highly warranted.

## Supporting information

S1 Fig COL6 does not enhance lung epithelial cell proliferation. Relative number of (a) 16HBE or (b) NHBE cells at 48 or 72 hrs post plating, quantified by MTT assay absorbance (570nm) cells. No significant differences were observed. These data are consistent with visual observations of proliferating cells. N = 9.
(DOCX)

S2 Fig COL6 does not enhance lung epithelial cell adhesion. Relative number of adherent cells measured at 2 hrs by MTT assay. No significant differences were observed. These data are consistent with visual observations of adherent cells. N = 9.
(DOCX)

S3 Fig Matrigel enhances lung fibroblast wound repair. (a) Representative images of wound-healing response for primary human lung fibroblasts plated on COL6, Matrigel, or COL1. (b) Quantitation of wound width at 10 hr post-injury (relative to 0 hr) for cells plated on individual matrices. N = 3, * p<0.05, ** p<0.01.
(DOCX)

S1 Text Methods: Cell adhesion and proliferation assays. (DOCX)

S2 Text Methods: Human lung fibroblast culture. (DOCX)

S1 Appendix Minimal underlying dataset. (ZIP)
