# Supplementary material for: Type VI collagen promotes lung epithelial cell spreading and wound-closure
Source: PLoS One. 2018 Dec 14;13(12):e0209095. doi: 10.1371/journal.pone.0209095 (PMC6294368; doi:10.1371/journal.pone.0209095)
Supplement: S1 Fig — Relative number of (a) 16HBE or (b) NHBE cells at 48 or 72 hrs post plating, quantified by MTT assay absorbance (570nm) cells. No significant differences were observed. These data are consistent with visual observations of proliferating cells. N = 9. (DOCX) [file pone.0209095.s001.docx]

b.

a.
