# Supplementary figures and images for: Type VI collagen promotes lung epithelial cell spreading and wound-closure
Source: PLoS One. 2018 Dec 14;13(12):e0209095. doi: 10.1371/journal.pone.0209095 (PMC6294368; doi:10.1371/journal.pone.0209095)

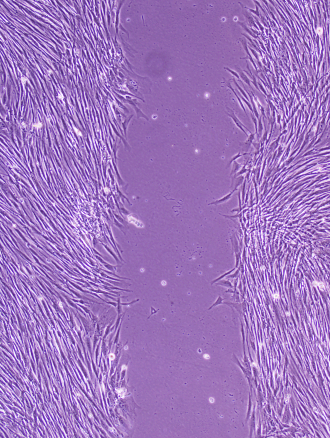

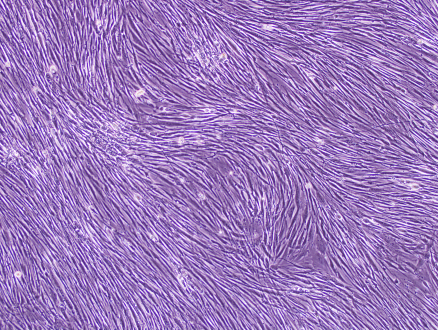

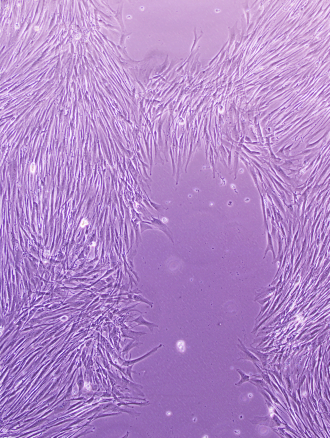

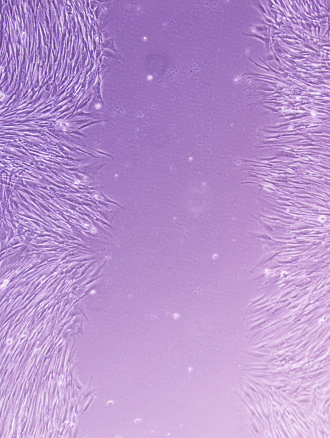

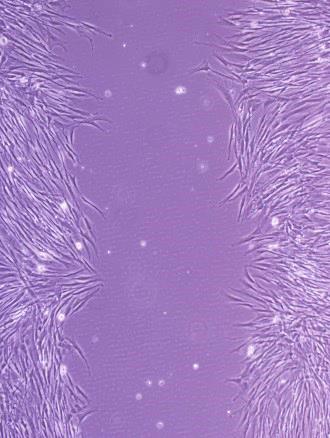

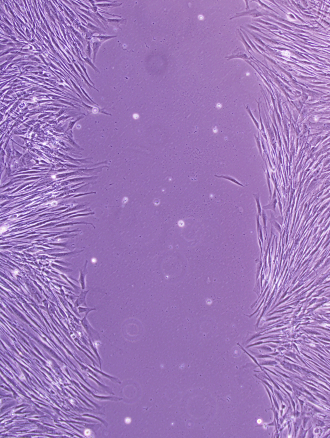


a.

n.s.

**

*

*

Collagen 1 Matrigel Collagen 6

0Hr 10Hr

Supplement: S3 Fig — (a) Representative images of wound-healing response for primary human lung fibroblasts plated on COL6, Matrigel, or COL1. (b) Quantitation of wound width at 10 hr post-injury (relative to 0 hr) for cells plated on individual matrices. N = 3, * p<0.05, ** p<0.01. (DOCX) [file pone.0209095.s003.docx]
