# Supplementary material for: Type VI collagen promotes lung epithelial cell spreading and wound-closure
Source: PLoS One. 2018 Dec 14;13(12):e0209095. doi: 10.1371/journal.pone.0209095 (PMC6294368; doi:10.1371/journal.pone.0209095)
Supplement: S1 Text — (DOCX) [file pone.0209095.s004.docx]

Cell Adhesion and Proliferation Assays

Flat-bottom 96-well plates were coated with COL6, COL1, or Matrigel as described with 20µl of diluted matrix solutions. 16HBE and NHBE cells were seeded at a density of 5000 cells per well. After 3hr for adhesion assays, or 48hr and 72hr for proliferation, media was removed and wells were washed with PBS. Live, adherent cells were quantified by Vibrant MTT Cell Proliferation Assay (Thermo Fisher, M6494). Absorbance of the formazan end product was measured at 570nm.
