# Supplementary material for: Type VI collagen promotes lung epithelial cell spreading and wound-closure
Source: PLoS One. 2018 Dec 14;13(12):e0209095. doi: 10.1371/journal.pone.0209095 (PMC6294368; doi:10.1371/journal.pone.0209095)
Supplement: S2 Text — (DOCX) [file pone.0209095.s005.docx]

Human Lung Fibroblast Culture

Human lung fibroblasts isolated from gentle trypsinization of initial PHLE cell cultures were cultured under standard conditions in 37°C in a humidified incubator containing 5% CO2, using DMEM (Gibco, 11965092) medium supplemented with 10% FBS (Gibco, 10082147), 1% penicillin/streptomycin (Gibco, 15140122), 1% nonessential amino acids (Gibco, 11140050), sodium pyruvate (Gibco, 11360070), and HEPES buffer (Gibco, 15630080). Media was replaced every 48 hours.
