# Supplementary material for: Type VI collagen promotes lung epithelial cell spreading and wound-closure
Source: PLoS One. 2018 Dec 14;13(12):e0209095. doi: 10.1371/journal.pone.0209095 (PMC6294368; doi:10.1371/journal.pone.0209095)
Supplement: Supplementary file 5 [file pone.0209095.s002.docx]

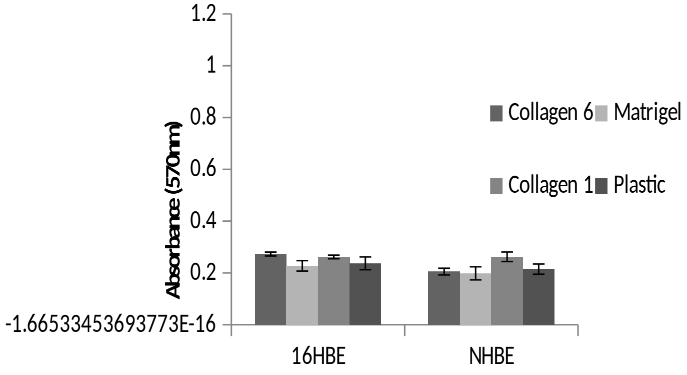

Absorbance (570nm)
1.2
1
0.8
0.6
0.4
0.2
-1.66533453693773E-16
16HBE
NHBE
Collagen 6
Matrigel
Collagen 1
Plastic
